# A follow-up study on the effect of exercise intervention on the executive functions of typical primary students and language learning difficulties

**DOI:** 10.3389/fpsyg.2026.1790230

**Published:** 2026-04-29

**Authors:** Haiyan Shi, Jiali Pan, Yinglun Tan, Chen Zeng, Lei Zhang

**Affiliations:** College of Physical Education and Health Science, Chongqing Normal University, Chongqing, China

**Keywords:** brain executive function, elementary school students with language learning difficulties, exercise Intervention, longitudinal study, regular elementary school student

## Abstract

**Objective:**

To reveal the effects of a developed “fun games + creative jump rope + creative running” exercise intervention program on the executive functions of elementary school students with Chinese language learning difficulties and their peers without such difficulties, revealing the causal relationship between exercise and executive function in elementary school students and its temporal characteristics.

**Methods:**

Forty-four students with Chinese language learning difficulties and 48 typically developing students were selected based on established criteria. The experimental group underwent a 10-week intervention using the developed “fun games + creative jump rope + creative running” program during physical education classes. Changes in executive function were assessed before the intervention, after 5 weeks, and after 10 weeks.

**Results:**

(1) The study found that the 10-week exercise intervention significantly impacted the executive function of both students with Chinese language learning difficulties and their typically developing peers. (2) The executive function levels of both groups after the 10-week intervention were higher than those after the 5-week intervention and higher than pre-intervention levels. (3) The improvement in executive function among students with Chinese language learning difficulties was greater than that among their typically developing peers following the 10-week intervention.

**Conclusion:**

The developed “fun games + fancy jump rope + fancy running” exercise intervention program positively impacts the executive function of both students with Chinese language learning difficulties and their typically developing peers. The improvement effect becomes more pronounced with increased intervention duration, and the experimental group of students with Chinese language learning difficulties demonstrated greater improvement in executive function than their typically developing counterparts.

## 1 Introduction

Executive function is one of the brain’s core functions and a crucial component of learning, reasoning, problem-solving, and intellectual activities (Funahashi, 2001).

The quality of executive function development not only impacts children’s physical and mental health but also influences their future social achievements. Research and exploration into methods promoting children’s executive function development have become a hot topic in academic studies. Continuous empirical research indicates that aerobic exercise not only enhances executive function development in typically developing children but also promotes it in children with special needs (e.g., obese children, children with hearing impairments and children with speech impairments, children with attention deficit hyperactivity disorder). This phenomenon is explained by theories such as the vascular change hypothesis, the cognitive neuroscience hypothesis, the cognitive reserve hypothesis, and the neurotrophic factor hypothesis (Liang et al., 2005; Chen and Yin, 2011; Chen et al., 2015; He, 1996; Lin, 2003; Shen, 1999; Stern, 2002; Stern, 2010; Yao and Li, 2006). Currently, aerobic exercise is recognized as a simple, effective, and low-cost means to enhance children’s executive function development.

Language learning difficulties refer to the phenomenon where students lag behind in language learning due to deficiencies in language proficiency, specifically falling significantly behind the level expected for their age or grade (Wang and Liu, 2008). Language serves as a communication tool and forms the foundation for learning other subjects. Language learning difficulties not only impact students’ academic performance across all subjects but also affect their physical and mental health, drawing attention from schools, parents, and society at large. Research indicates that deficits in working memory capacity are a significant cause of language learning difficulties (Li et al., 2011; Luo, 2012; Wang, 2007). Therefore, promoting improvements in executive function—particularly working memory—among elementary students with language-learning difficulties and thereby enhancing their academic performance has become critically important. To date, no longitudinal studies have examined the effects of physical exercise on the executive functions of both language-learning-impaired and typically developing elementary students. Based on this gap, this study developed a “fun games + creative jump rope + creative running” exercise intervention program. This program targets the underdeveloped working memory function in language-learning-impaired students and is grounded in the theory that physical activity enhances children’s executive functions. This 10-week intervention program was implemented during physical education classes to investigate the effects of the developed “fun games + creative jump rope + creative running.” An exercise regimen on the executive functions of both students with language learning difficulties and their typically developing peers. The study aims to reveal the causal relationship between physical activity and executive function development in elementary students, as well as the temporal characteristics of these effects, thereby providing a practical foundation for enhancing executive function development in students with language learning difficulties.

## 2 Research subjects and methods

### 2.1 Research subjects

#### 2.1.1 Selection of elementary students with Chinese language learning difficulties

Following previous domestic studies (Li and Bai, 2008; Wang and Liu, 2007), the selection criteria for elementary students with Chinese language learning difficulties were: ➀ typical intelligence with an IQ > 90, as determined by the Raven’s Standard Progressive Matrices (Zhang and Wang, 1989); ➁ Final Chinese language exam scores below 70; ➂ Teacher-identified Chinese language learning difficulties.

#### 2.1.2 Experimental grouping

Two natural class groups were selected, with one randomly assigned as the experimental group and the other as the control group. Based on the selection criteria, 44 students with Chinese language learning difficulties were divided into an experimental group (*n* = 24) and a control group (*n* = 20). Additionally, 48 typically developing students were divided into an experimental group (*n* = 23) and a control group (*n* = 25). The experimental group received physical intervention during physical education classes using the developed “Fun Games + Creative Jump Rope + Creative Running” exercise program, while the control group continued with regular learning and daily activities.

### 2.2 Research methods

#### 2.2.1 Content of the exercise intervention program

The developed “Fun Games + Creative Jump Rope + Creative Running” exercise intervention program includes exercise activities (Fun Games, Creative Jump Rope, Creative Running); exercise intensity [moderate intensity: (220–age) × (60–69%)]; exercise frequency (3 times per week); and exercise duration (30 min per session) (see Table 1).

**TABLE 1:** Overview of exercise intervention program components: primary exercise content.

Primary exercise content
Week 1: Instructor guides students through fun games: heel-to-toe jumps (forward/backward, left/right); combination jump 1. Students learn foot-flexibility exercises, then fast running, followed by short-stride running, then fast running again.
Week 2: Teacher guides students in fun games; students learn jumping jacks (forward/backward, left/right), combination jump 2; students learn short-step running followed by fast running, high-knee running followed by fast running.
Week 3: Teacher guides students in fun games; students learn combination jump 3, combination jump 4; students learn back-kick running, followed by fast running, figure-eight running.
Week 4: Teacher guides students in fun games; Students learn single-leg hops, alternating-leg hops; Students learn figure-eight running, obstacle course challenge.
Week 5: Teacher guides students in fun games; Students learn left-knee-up hops, alternating left/right knee-up hops, right-knee-up hops; Students learn variable-speed running, figure-eight running.
Week 6: Teacher guides students in fun games; Students learn alternating knee lifts, combination jump 5; students learn figure-eight running, agility ladder 1.
Week 7: Teachers guide students in fun games; students learn inward snap kicks, inward/outward snap kicks, outward snap kicks; students learn agility ladder 2, agility ladder 3.
Week 8: Teachers guide students in fun games; students learn combination jump 6 and group pattern jumps; students learn agility ladder exercises 3 and 4.
Week 9: Teachers guide students in fun games; students learn stationary long-rope jumping and moving long-rope jumping; students learn combination running patterns 1 and 2.
Week 10: Teachers guide students in fun games; students learn moving figure-eight long rope jumping; students learn combination running pattern 2 and the “Bravery Challenge.”

#### 2.2.2 Executive function assessment

The Executive Function Assessment Tool for Children, developed by Chen et al. (2011), was used to assess inhibitory control, working memory, and flexible shifting abilities in elementary students with language-learning difficulties. This was achieved through the Flank Task, 1-back Task, and More-Odd Shifting Task.

#### 2.2.3 Experimental procedure

A 2-group (experimental group, control group) × 2-type (typical elementary students, language-impaired elementary students) × 3-time (pre-test, mid-test, post-test) mixed design was employed. The experiment comprised a pre-test, the implementation of the “fun games + fancy jump rope + fancy running” exercise intervention, a mid-test, the continuation of the intervention, and a post-test. The pre-test was conducted 1 week before the intervention; the mid-test occurred 5 weeks after the exercise intervention; and the post-test was administered 10 weeks after the exercise intervention.

#### 2.2.4 Statistics and analysis

Executive function data were processed and analyzed using SPSS 21.0. The statistical method employed repeated measures analysis of variance to examine the individual and interactive effects of three factors: group, type, and time. When three-way interactions were significant, simple effects analysis was conducted for further investigation. *p* < 0.05 indicates a statistically significant difference.

## 3 Results and analysis

### 3.1 Descriptive statistics of executive function in elementary students with language learning difficulties and typically developing students before, during, and after the exercise intervention

Table 2 shows that, in terms of executive function trends, the intervention group of elementary school students with language learning difficulties demonstrated sustained improvements in inhibition, working memory, and shifting from the pre-test to the mid-test and post-test. Reaction times gradually decreased, and cognitive performance steadily improved; in contrast, the control group showed no significant changes in reaction times for inhibition, working memory, and shifting. Among typically developing elementary school students, the intervention group also demonstrated a sustained and stable upward trend in inhibition, working memory, and shifting, with reaction times gradually decreasing throughout the testing process and cognitive efficiency continuously improving; the control group showed no significant changes in reaction times for these three functions.

**TABLE 2:** Executive function reaction times in the experimental and control groups before, during, and after testing.

Types of students	Subfunctions of executive function	Experimental group (M ± SD)			Control group (M ± SD)		
		Pre-test	Mid-test	Post-test	Pre-test	Mid-test	Post-test
Typical elementary school student	Inhibition	14.32 ± 33.56	9.61 ± 25.37	7.52 ± 15.76	16.25 ± 41.66	15.20 ± 30.38	13.10 ± 34.95
	Working memory	900.13 ± 136.42	756.45 ± 117.76	672.92 ± 79.40	936.69 ± 102.47	900.03 ± 78.49	876.01 ± 69.14
	Switch	424.97 ± 86.50	390.72 ± 112.23	381.89 ± 102.79	455.69 ± 110.02	433.24 ± 83.26	432.33 ± 89.78
Language-impaired elementary school student	Inhibition	20.76 ± 30.09	17.76 ± 14.76	14.34 ± 28.34	6.917 ± 29.43	10.98 ± 24.70	16.21 ± 25.94
	Working memory	1098.09 ± 258.87	767.64 ± 201.71	682.92 ± 123.54	1057.03 ± 129.38	1024.53 ± 116.80	1015.02 ± 164.77
	Switch	485.24 ± 173.91	447.75 ± 89.53	427.29 ± 77.69	485.80 ± 59.92	481.35 ± 54.81	478.27 ± 52.13

### 3.2 Executive function characteristics of elementary students with language learning difficulties

A *t*-test was conducted comparing the executive functions of students with language learning difficulties and their typically developing peers. Table 3 shows that students with language-learning difficulties exhibited significantly lower working memory capacity than those without such difficulties (*p <* 0.05). In contrast, inhibitory control and flexible switching abilities did not differ significantly between these two groups (*P* > 0.05). Overall, the study indicates impaired working memory development in elementary students with language learning difficulties.

**TABLE 3:** *T*-test results for developmental characteristics of executive function in Chinese language-impaired and typically developing elementary students (M ± SD).

Sub-function	Students with language difficulties	Regular elementary school student	*t*	*p*
Inhibition	14.47 ± 30.26	15.32 ± 37.61	−1.20	0.905
Working memory	1079.43 ± 208.97	919.17 ± 120.06	4.56	0.000**
Shifting	485.49 ± 133.28	440.97 ± 99.62	1.82	0.07

*p < 0.05;

**p < 0.01.

### 3.3 Homogeneity test for executive functions

#### 3.3.1 Homogeneity test for pre-intervention executive functions in elementary students with language learning difficulties

A one-way ANOVA was conducted on executive functions in elementary students with language-learning difficulties in the experimental and control groups prior to the motor intervention. Results indicated: Inhibition function [*F*(1, 43) = 2.35, *P* = 0.132 > 0.05], reset function [*F*(1, 43) = 0.415, *P* = 0.523 >0.05], and switching function [*F*(1, 43) = 0.000, *P* = 0.989>0.05]. No significant differences existed among the sub-functions of executive function (inhibition, reset, and switching), indicating that the levels of executive function among elementary students with language learning difficulties were homogeneous prior to the physical intervention.

Table 2 shows that, in terms of trends in executive function, among elementary school students with reading difficulties, the intervention group demonstrated continuous improvement in inhibitory control, updating, and shifting from the pre-test to the mid-test and then to the post-test. Reaction times gradually decreased, and cognitive abilities steadily improved as the intervention progressed; In contrast, the control group showed no significant improvement; in fact, inhibitory function even regressed, while the other two functions exhibited only slight fluctuations. Among typically developing elementary students, the intervention group’s inhibitory, updating, and shifting functions similarly demonstrated a sustained and steady upward trend, with reaction times gradually decreasing throughout the testing process and cognitive efficiency continuously improving; the control group s three functions showed only minor improvements, with the magnitude far smaller than that of the intervention group, and did not establish a stable upward trend.

Table 3 shows that Independent-samples *t*-tests revealed that children with Chinese language learning difficulties exhibited comparable inhibition but significantly superior working memory performance compared to typically developing peers, while a marginal trend favored the typical peers in shifting function.

#### 3.3.2 Homogeneity test for pre-intervention executive function measures in typical elementary school students

A one-way ANOVA was conducted on the sub-functions of executive function for both the experimental and control groups of typical elementary school students prior to the motor intervention. Results indicated: No significant differences were found in inhibitory function [*F*(1, 47) = 0.031, *P* = 0.861>0.05], and switching function [*F*(1, 47) = 1.143, *P* = 0.291>0.05]. No significant differences were observed among the executive function subfunctions (inhibition, working memory, and shifting), indicating homogeneity in executive function levels among typical elementary students prior to the physical intervention.

### 3.4 Follow-up analysis of motor intervention effects on executive function in students with language learning difficulties and typical students

#### 3.4.1 Tracking the effects of exercise intervention on executive function in elementary students with language difficulties

##### 3.4.1.1 Effects on inhibitory function at three time points

Due to the triple interaction of time × group × type, simple effects analysis revealed:

(1) Intergroup comparison of inhibitory function in elementary students with Chinese language learning difficulties at three time points

**FIGURE 1:**
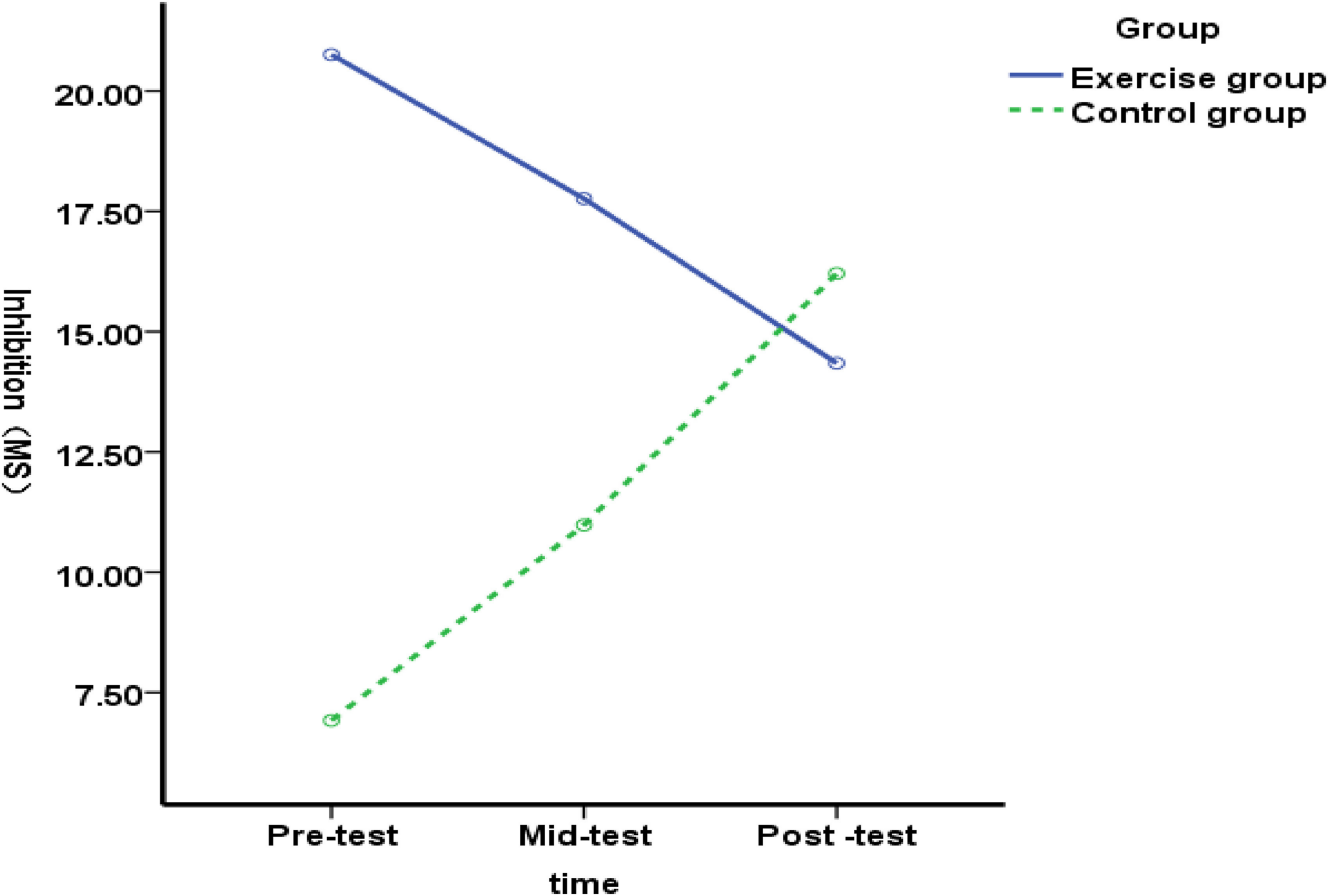
Changes in inhibitory function among elementary students with language learning difficulties before, during, and after exercise intervention.

We compared inhibitory function between the experimental and control groups at pre-test (before the exercise intervention), mid-test (after 5 weeks), and post-test (after 10 weeks) in elementary students with Chinese language learning difficulties. At pre-test, no significant difference was found between the groups [*F*(1, 89) = 1.76, *p* = 0.187 > 0.05], indicating similar inhibitory function before intervention. At mid-test (after 5 weeks), results still showed no significant difference [*F*(1, 89) = 0.89, *p* = 0.347 > 0.05]. This suggests the 5-week exercise intervention did not affect inhibitory function. After 10 weeks (post-test), there was still no significant difference [*F*(1, 89) = 0.03, *p* = 0.867 > 0.05], indicating the longer exercise intervention (5–10 weeks) also had no effect. These findings are shown in Figure 1. In summary, neither the 5-week nor the 10-week exercise interventions affected the inhibitory function of elementary students with Chinese language learning difficulties.

(2) Intragroup multiple comparisons of inhibitory function at three time points

No significant differences in inhibitory function were observed among the experimental group of elementary students with language learning difficulties before, during, and after the intervention [*F*(2, 176) = 0.30, *p* = 0.744, > 0.05]. The results indicate that although the improvement in inhibitory function among elementary students with language learning difficulties was not statistically significant, a trend toward improvement was observed as the duration of the exercise intervention increased.

##### 3.4.1.2 Effects of working memory function on elementary students with language difficulties at three time points

Due to the triple interaction of time × group × type, simple effects analysis revealed:

(1) Intergroup comparison of working memory function in elementary students with Chinese language learning difficulties at three time points

**FIGURE 2:**
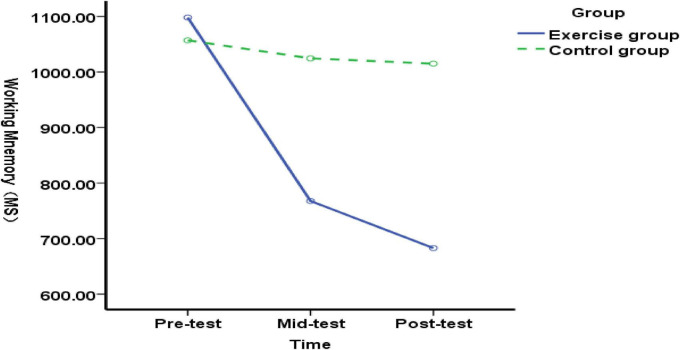
Changes in working memory function in elementary students with language learning difficulties before, during, and after exercise intervention.

We compared the working memory function in experimental and control groups of elementary students with Chinese language learning difficulties. Measurements were taken at pre-test (before exercise intervention), mid-test (after 5 weeks), and post-test (after 10 weeks). At pre-test, no significant difference in working memory function was found [*F*(1, 89) = 0.99, *p* = 0.322, *P*>0.05], indicating that both groups had similar working memory function before the intervention. At the mid-test (end of 5-week exercise intervention), a highly significant difference was found in the working memory function between the experimental and control groups of elementary students with Chinese language learning difficulties [*F*(1, 89) = 34.89, *p* = 0.000<0.01], indicating that the 5-week exercise intervention positively impacted the working memory function of these students; Post-test (after 10 weeks of exercise intervention): The working memory function of elementary students with Chinese language learning difficulties in the experimental and control groups showed a highly significant difference [*F*(1, 89) = 82.60, *p* = 0.000 < 0.01], indicating that the 5–10 week exercise intervention positively impacted the working memory function of these students (Figure 2). The results indicate that both 5-week and 10-week exercise interventions improved working memory in elementary students with Chinese language learning difficulties.

(2) Intra-group multiple comparisons of working memory function at three time points for elementary students with Chinese language learning difficulties

The experimental group’s working memory function for elementary students with Chinese language learning difficulties showed highly significant differences across the pre-, mid-, and post-tests [*F*(2, 176) = 54.95, *p* = 0.000 < 0.01]. Therefore, a Bonferroni multiple-comparison analysis was applied. As shown in Table 4, the mean difference between the pre-test and mid-test working memory functions was 330.452, *p* = 0.001<0.01, indicating that the mid-test working memory function outperformed the pre-test with significant difference; The mean difference between the pre-test and post-test for recall function was 415.168, *p* = 0.000<0.01, indicating that the post-test recall function was superior to the pre-test and showed a significant difference; the mean difference between the mid-test and post-test for recall function was 84.716, *p* = 0.157>0.05, indicating that the post-test recall function was superior to the mid-test but without a significant difference.

**TABLE 4:** Intra-group multiple comparisons of brushing function in elementary students with Chinese language learning difficulties at three time points.

(I) Time	(J) Time	Mean difference (I-J)	Standard error	*P*
Pre-test	Mid-test	330.452	80.915	0.001**
	Post-test	415.168	60.713	0.000**
Mid-test	Pre-test	−330.452	80.915	0.001**
	Post-test	84.716	41.407	0.157
Post-test	Pre-test	−415.168	60.713	0.000**
	Mid-test	−84.716	41.407	0.157

Raw data with significant mean differences are marked with “**”.

Results indicate that the working memory effect on elementary students with language-learning difficulties increases more effectively as the duration of the motor intervention increases.

As can be seen from Table 5, A three-way repeated-measures ANOVA was conducted to examine the motor intervention effects on executive function in students with language difficulties and typically developing students. For the inhibition function, no significant main effects or interactions were observed for group, student type, time, or their combinations (*p* > 0.05), with negligible effect sizes (η^2^). For working memory, the main effects of group, student type, and time were all highly significant (*p* < 0.01), with a large effect size for the time main effect (η^2^ = 0.268); the time × interaction was highly significant (*p* < 0.01), and the time × group × type interaction was significant (*p* < 0.05), while other interactions were not significant. For the shifting function, the main effects of group and student type were significant (*p* < 0.05); the time main effect was marginally significant (*p* = 0.038, η^2^ = 0.058); and all interaction effects were non-significant (*p* > 0.05).

**TABLE 5:** Analysis of variance for motor intervention effects on executive function in students with language difficulties and typical students.

Function	Interaction	Type III sum of squares	*df*	Mean square	*F*	*p*	η^2^
Inhibition	Group	60.955	1	60.955	0.071	0.791	0.01
	Type	228.764	1	228.764	0.265	0.608	0.03
	Time	147.745	2	73.873	0.089	0.915	0.001
	Group × Type	1931.872	1	1931.872	2.242	0.138	0.007
	Time × Group	1073.331	2	536.666	0.643	0.527	0.025
	Time × Type	470.112	2	235.056	0.282	0.755	0.06
	Time × Group × Type	441.087	2	220.543	0.264	0.768	0.003
Working memory	Group	1650161.709	1	1650161.709	91.334	0.000**	0.00
	Type	692002.536	1	692002.536	38.301	0.000**	0.00
	Time	1695742.456	2	847871.228	40.340	0.000**	0.268
	Group × Type	51637.924	1	51637.924	2.858	0.094	0.00
	Time × Group	901023.062	2	450511.531	21.435	0.000**	0.00
	Time × Type	118344.391	2	59172.196	2.815	0.063	0.00
	Time × Group × Type	151099.734	2	75549.867	3.595	0.030*	0.00
Shifting	Group	82987.661	1	82987.661	6.603	0.012*	0.00
	Type	156594.250	1	156594.250	12.459	0.001**	0.00
	Time	53751.450	2	26875.725	3.345	0.038*	0.058
	Group × Type	2827.422	1	2827.422	0.225	0.636	0.00
	Time × Group	14403.515	2	7201.757	0.896	0.410	0.00
	Time × Type	780.690	2	390.345	0.049	0.953	0.00
	Time × Group × Type	2820.987	2	1410.493	0.176	0.839	0.00

*p < 0.05; **p < 0.01.

**FIGURE 3:**
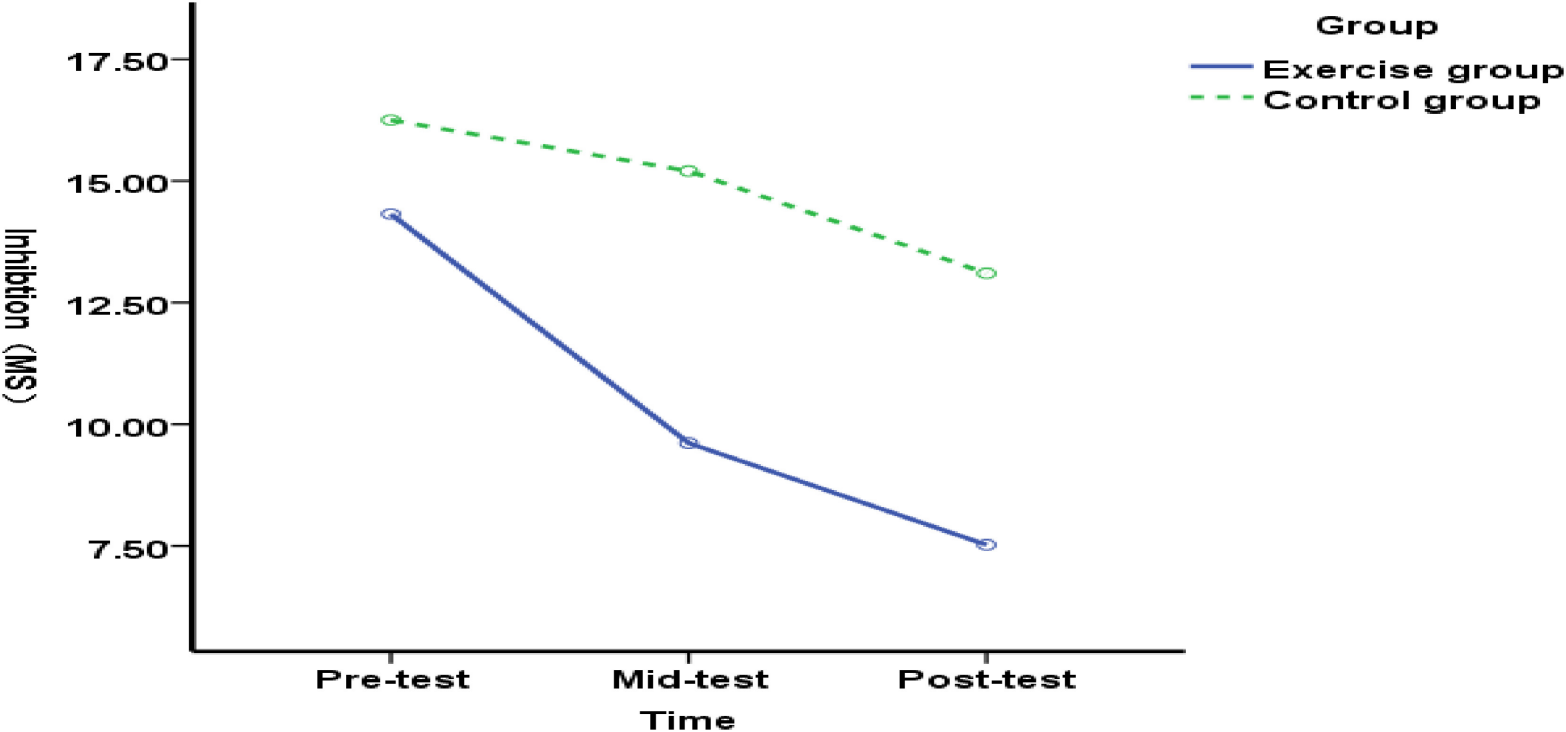
Changes in inhibitory function among typical elementary school students before, during, and after the exercise intervention.

The three-way interaction analysis (Time × Group × Type) in Figure 3 revealed that the interaction effect on the inhibition function was not statistically significant (*F* = 0.264, *p* = 0.768, η^2^ = 0.003); The interaction effect for the working memory function was significant (*F* = 3.595, *p* = 0.030, η^2^ = 0.00), suggesting that time, group, and student type interact to influence working memory performance; the interaction effect for the shifting function was also not significant (*F* = 0.176, *p* = 0.839, η^2^ = 0.00).

##### 3.4.1.3 Effects of functional shifting in elementary students with language difficulties across three time points

Due to the triple interaction of time × group × type, simple effects analysis revealed:

(1) Intergroup comparisons of shifting function in elementary students with Chinese language learning difficulties across three time points

**FIGURE 4:**
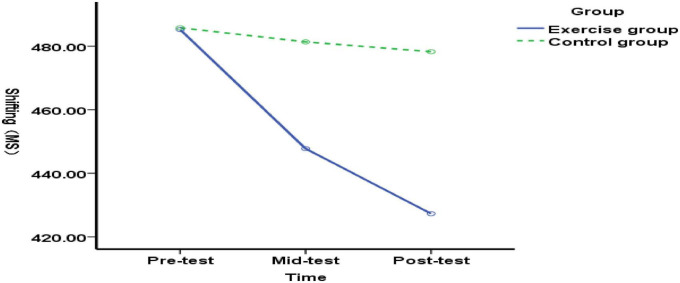
Changes in transitional functions among elementary students with language difficulties before, during, and after the exercise intervention.

Comparing differences in shifting function between the experimental and control groups at pre-test (before exercise intervention), mid-test (after 5 weeks of exercise intervention), and post-test (after 10 weeks of exercise intervention): No significant difference existed in shifting function between the experimental and control groups at pre-test [*F*(1, 89) = 0.01, *P* = 0.918>0.05], indicating homogeneous transfer function between the experimental and control groups before the exercise intervention; At the mid-test (end of 5 weeks of exercise intervention), no significant difference was found in shifting function between the experimental and control groups of elementary students with Chinese language learning difficulties [*F*(1, 89) = 1.07, *P* = 0.305>0.05], indicating that the 5-week exercise intervention had no effect on shifting function in these students; Post-test (after 10 weeks of exercise intervention): No significant difference in shifting function between the experimental and control groups of Chinese language learning-impaired elementary students [*F*(1, 89) = 3.19, *p* = 0.077 > 0.05], indicating that 5–10 weeks of exercise intervention had no effect on shifting function in these students (Figure 4). The results indicate that both 5- and 10-week exercise interventions did not affect the shifting function of elementary students with Chinese language learning difficulties.

(2) Intra-group multiple comparisons of functional shifting in elementary students with language difficulties at three time points

There was no significant difference in the shifting function of the experimental group of elementary students with language learning difficulties before, during, and after the intervention [*F*(2, 176) = 2.58, *p* = 0.079 > 0.05]. The results indicate that although the improvement in shifting function among elementary students with language learning difficulties was not statistically significant as the duration of the exercise intervention increased, a trend toward improvement was observed.

#### 3.4.2 Tracking the effects of exercise intervention on executive function in typically developing elementary school students

##### 3.4.2.1 The effects of inhibitory functions on typical elementary school students at three time points

Due to the triple interaction of time × group × type, a simple effect test revealed:

(1) Intergroup comparison of typical elementary school students’ inhibitory function at three time points

Comparing inhibitory function differences between the experimental and control groups at pre-test (before exercise intervention), mid-test (end of 5-week exercise intervention), and post-test (end of 10-week exercise intervention) among typically developing elementary students: No significant difference in inhibitory function was observed between the experimental and control groups at pre-test [*F*(1, 89) = 0.04, *p* = 0.841>0.05], indicating homogeneous inhibitory function between the groups prior to the exercise intervention. At the mid-test (end of 5 weeks of exercise intervention), no significant difference in inhibitory function was observed between the experimental and control groups of typical elementary school students [*F*(1, 89) = 0.61, *p* = 0.436>0.05], suggesting that the 5-week exercise intervention had no effect on inhibitory function in typical elementary school students. Post-test (after 10 weeks of exercise intervention) showed no significant difference in inhibitory function between the experimental group and the control group of typical elementary school students [*F*(1, 89) = 0.46, *p* = 0.497 > 0.05], indicating that the 5–10-week exercise intervention had no effect on the inhibitory function of typical elementary school students (Figure 3). The results indicate that both the 5- and 10-week exercise interventions had no effect on the inhibitory function of typical elementary school students.

(2) Intragroup multiple comparisons of inhibitory function in typical elementary school children at three time points

There was no significant difference in inhibitory function among pre-, mid-, and post-tests in the experimental group of typical elementary school students [*F*(2, 176) = 0.33, *p* = 0.716 > 0.05]. The results indicate that although the improvement in inhibitory function among typical elementary school students was not statistically significant, a trend toward improvement was observed as the duration of the exercise intervention increased.

##### 3.4.2.2 Impact of the working memory function on typical elementary school students at three time points

Due to the triple interaction of time × group × type, a simple effect test revealed that:

(1) Intergroup comparison of typical elementary school students’ working memory function at three time points

**FIGURE 5:**
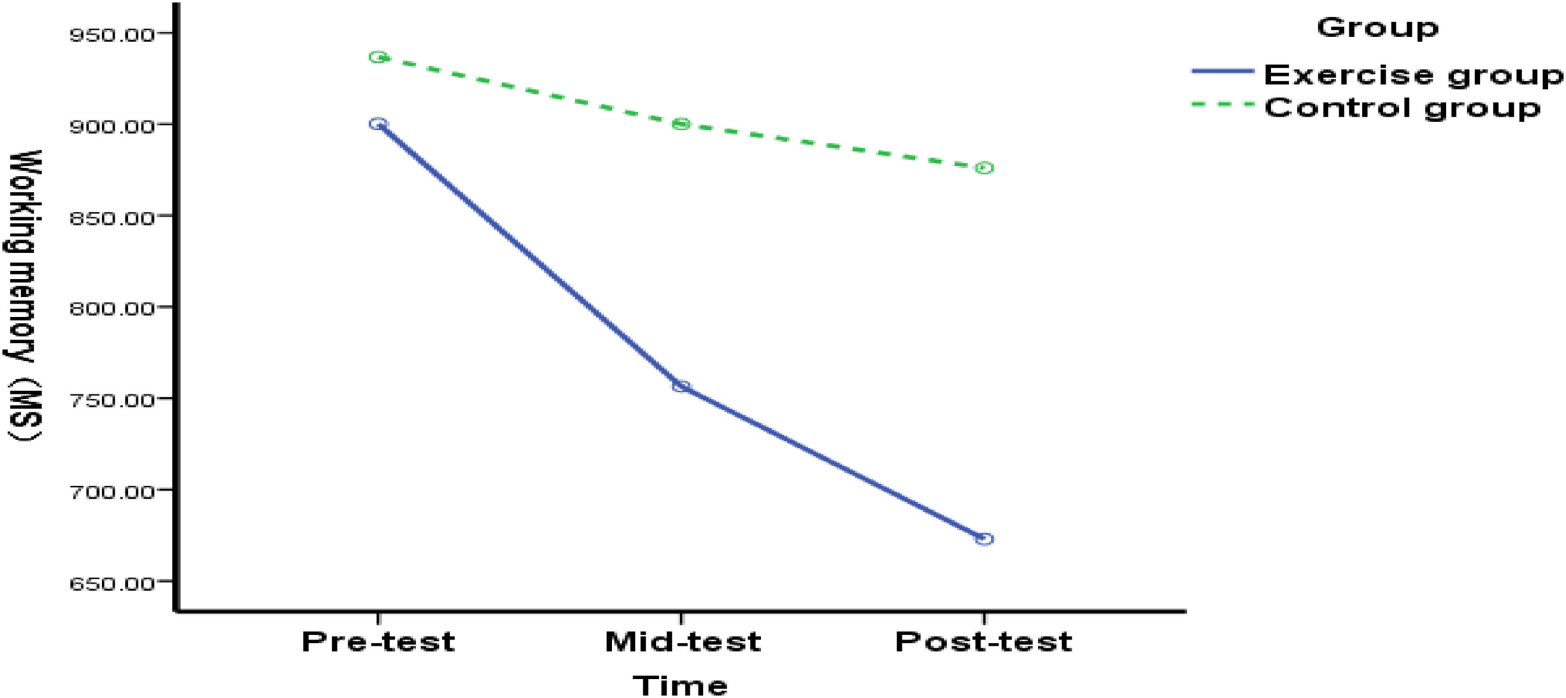
Changes in working memory function in typical elementary school students before, during, and after exercise intervention.

Comparing differences in typical elementary students’ working memory function between the experimental and control groups at pre-test (before exercise intervention), mid-test (after 5 weeks of exercise intervention), and post-test (after 10 weeks of exercise intervention):No significant difference in working memory function was found between the experimental and control groups at pre-test [*F*(1, 89) = 0.31, *p* = 0.576>0.05], indicating homogeneous working memory functions prior to the exercise intervention. At the mid-test (end of 5 weeks of exercise intervention), a highly significant difference was observed in working memory function between the experimental and control groups [*F*(1, 89) = 12.08, *p* = 0.001<0.01], indicating that the 5-week exercise intervention positively impacted working memory function in typical elementary school students; Post-test (after 10 weeks of exercise intervention) revealed a highly significant difference in working memory function between the experimental and control groups [*F*(1, 89) = 34.27, *p* = 0.000 < 0.01], indicating that the 5–10 week exercise intervention positively impacted working memory function in typical elementary students (Figure 5). The results indicate that both 5-week and 10-week exercise interventions exerted positive effects on the working memory function of typical elementary school students.

(2) Multiple comparisons within groups for the typical elementary school student working memory function at three time points

The experimental group of typical elementary school students exhibited highly significant differences in recall function across pre-, mid-, and post-tests [*F*(2, 176) = 14.45, *p* = 0.000 < 0.01].Therefore, multiple comparisons were further analyzed using the Bonferroni method. As shown in Table 6, the mean difference between the pre-test and mid-test working memory function scores was 143.682 (*p* = 0.002<0.01), indicating that mid-test working memory function performance was superior to pre-test performance and was significantly different. The mean difference between the pre-test and post-test for the working memory function was 227.214 (*p* = 0.000<0.01), indicating that post-test performance was superior to pre-test performance and that the difference was significant. The mean difference between the mid-test and post-test for the working memory function was 83.532 (*p* = 0.55>0.05), indicating that post-test performance was superior to mid-test performance but without a significant difference.

**TABLE 6:** *Post-hoc* multiple comparisons within groups for typical elementary students’ working memory function at three time points.

(I) Time	(J) Time	Mean difference (I-J)	Standard error	*P*
Pre-test	Mid-test	143.682	37.134	0.002**
	Post-test	227.214	29.037	0.000**
Mid-test	Pre-test	−143.682	37.134	0.002**
	Post-test	83.532	32.782	0.55
Post-test	Pre-test	−227.214	29.037	0.000**
	Mid-test	−83.532	32.782	0.55

Raw data with significant mean differences are marked with “**”.

Results indicate that the working memory function of typical elementary school students improves more effectively with increased duration of motor intervention.

##### 3.4.2.3 The impact of three time points on typical elementary students’ shifting function

Due to the triple interaction of time × group × type, a simple effect test revealed:

(1) Intergroup comparison of typical elementary school students’ shifting function at three time points

**FIGURE 6:**
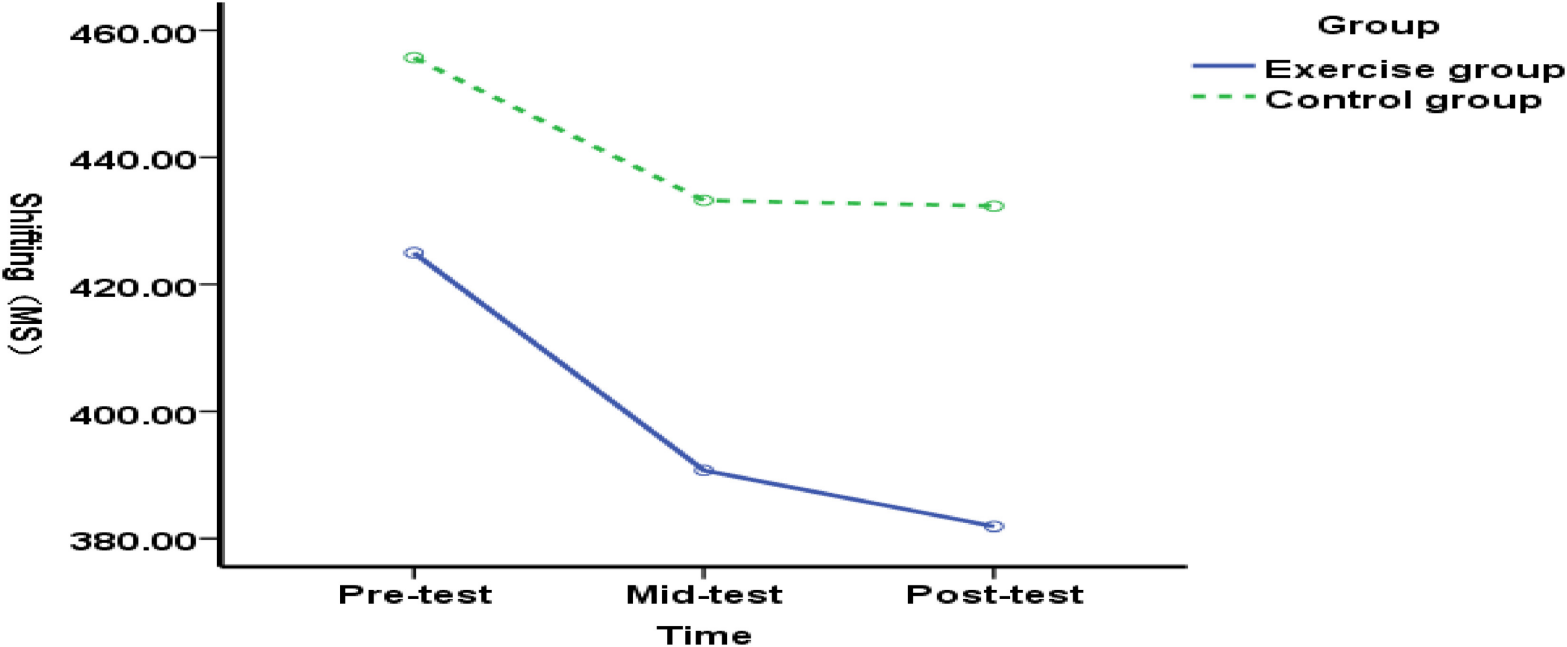
Changes in transitional function in typical elementary school students before, during, and after exercise intervention.

Comparing the differences in shifting function between the experimental group and the control group at pre-test (before exercise intervention), mid-test (end of 5 weeks of exercise intervention), and post-test (end of 10 weeks of exercise intervention) among typical elementary school students: There was no significant difference in shifting function between the experimental group and the control group at pre-test [*F*(1, 89) = 0.71, *p* = 0.403> 0.05], indicating homogeneous transfer function between the experimental and control groups before the exercise intervention. At the mid-test (end of 5 weeks of exercise intervention), no significant difference was found in transfer function between the experimental and control groups of typical elementary school students [*F*(1, 89) = 2.33, *p* = 0.130> 0.05], suggesting that the 5-week exercise intervention had no effect on transfer function in typical elementary school students. Post-test (after 10 weeks of exercise intervention) revealed no significant difference in shifting function between the experimental and control groups of typical elementary students [*F*(1, 89) = 3.80, *p* = 0.054 > 0.05], indicating that the 5–10-week exercise intervention had no effect on shifting function in typical elementary students (Figure 6). The results demonstrate that both 5-week and 10-week exercise interventions had no impact on shifting function in typical elementary students.

(2) Multiple comparisons within groups for typical elementary school children’s shifting function at three time points

There was no significant difference in the shifting function of the experimental group of typical elementary school students before, during, and after the intervention [*F*(2, 176) = 1.48, *p* = 0.230 > 0.05]. The results indicate that although the improvement in shifting function among typical elementary school students was not statistically significant, a trend toward improvement was observed as the duration of the exercise intervention increased.

#### 3.4.3 Tracking the effects of exercise intervention on executive function in Chinese-language struggling and typically developing elementary students in the experimental group

##### 3.4.3.1 Comparison of inhibitory functions between experimental group students with language difficulties and typically developing elementary students at three time points

**FIGURE 7:**
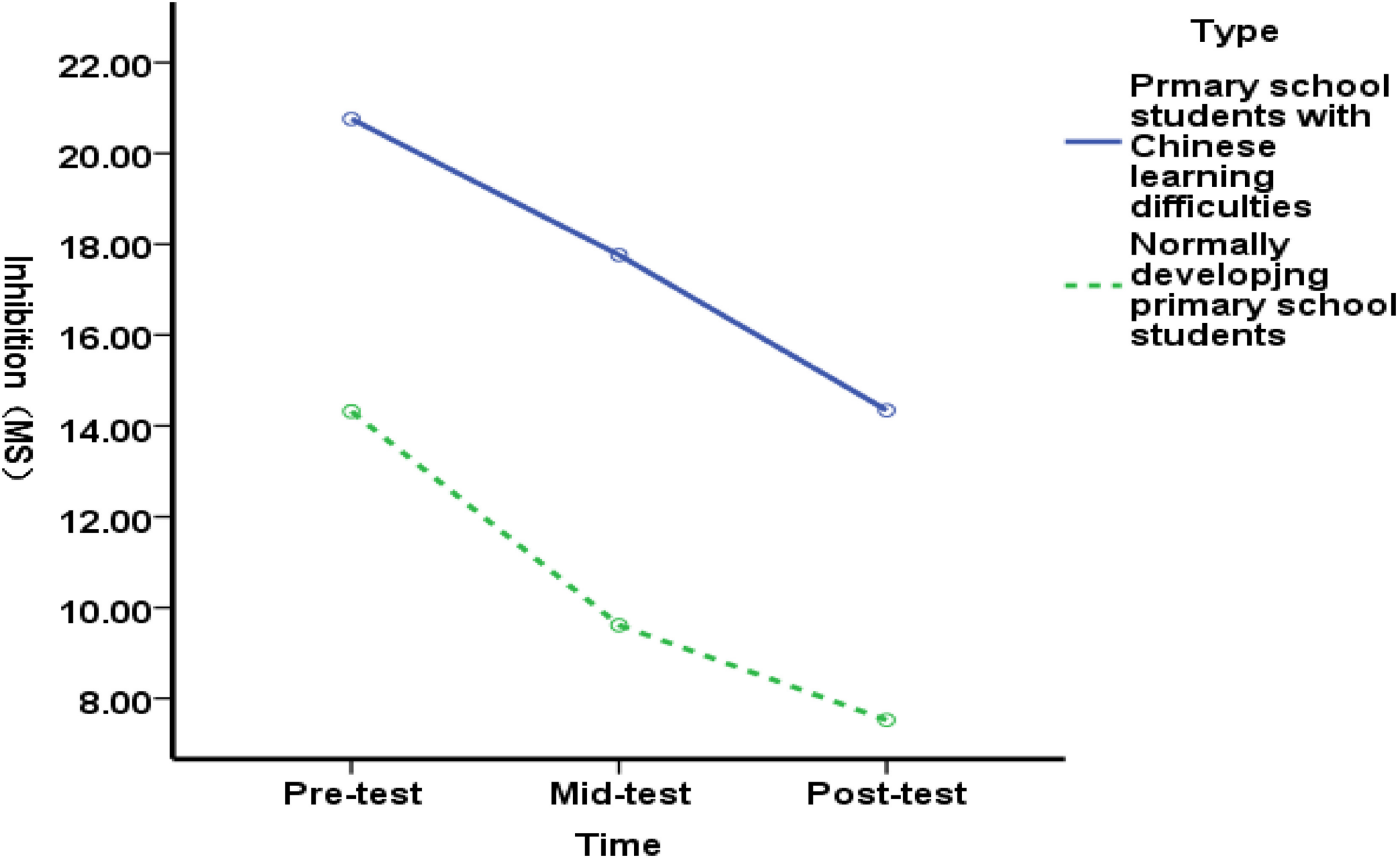
Changes in inhibitory function among Chinese language-impaired and typically developing elementary students before, during, and after the exercise intervention.

Figure 7 shows that, Pre-test comparisons revealed no significant difference in inhibitory function between the experimental group with language difficulties and the experimental group without language difficulties [*F*(1, 89) = 0.43, *p* = 0.513>0.05], indicating that both groups exhibited comparable inhibitory function levels. At the mid-test, no significant difference was found between the inhibitory function of the experimental group with language difficulties and that of the experimental group without language difficulties [*F*(1, 89) = 1.32, *p* = 0.254>0.05]; At the post-test, no significant difference was found between the inhibitory function of the experimental group with language difficulties and that of the experimental group without language difficulties [*F*(1, 89) = 0.72, *p* = 0.399>0.05]. Results indicate that the 10-week exercise intervention did not improve inhibitory function in either the experimental group with language difficulties or the typically developing elementary students.

##### 3.4.3.2 Comparison of working memory functionality between experimental group students with language difficulties and typically developing elementary students at three time points

**FIGURE 8:**
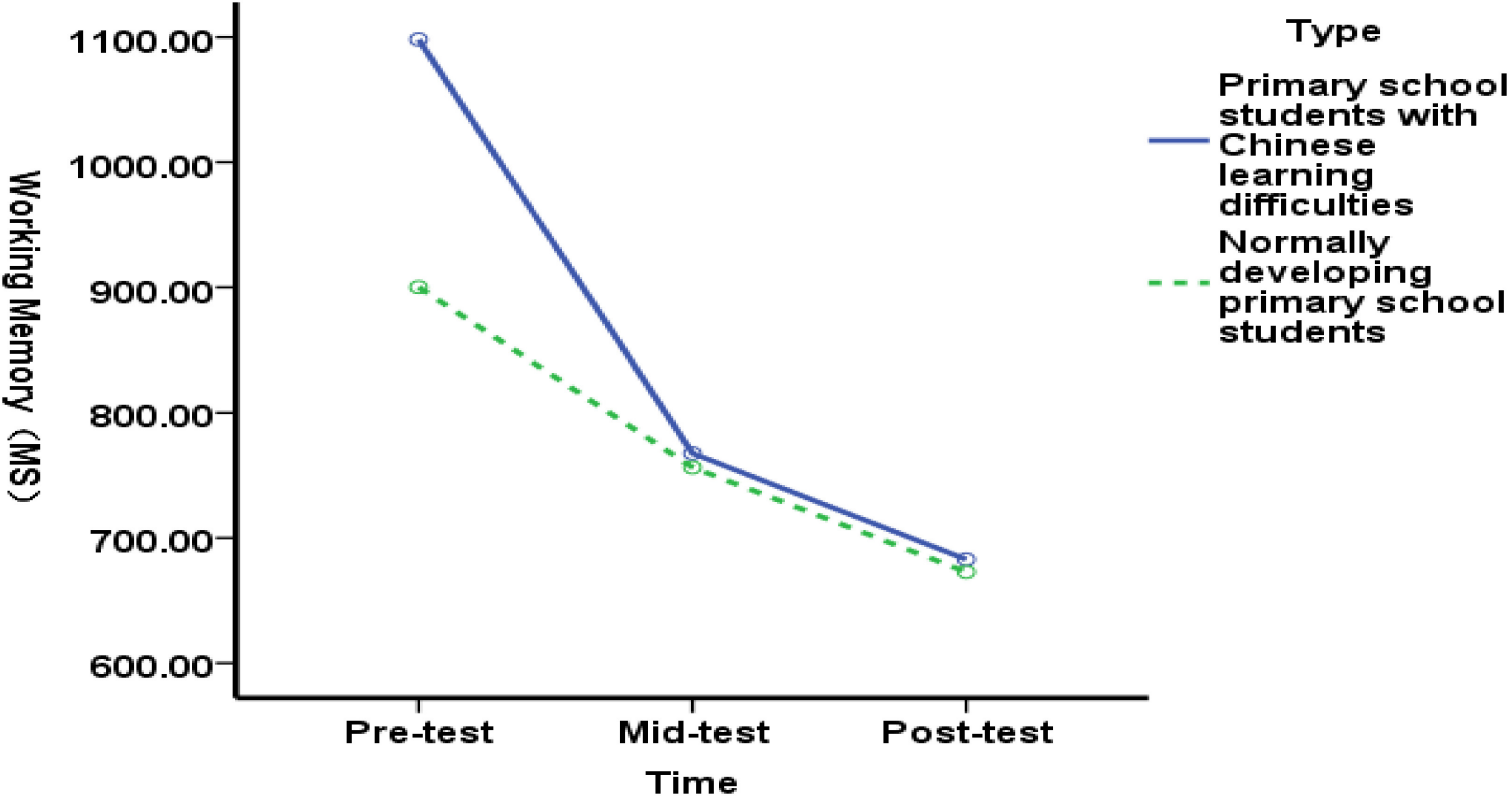
Changes in working memory function in students with language difficulties and typically developing elementary students before, during, and after the exercise intervention.

Pre-test comparisons revealed a highly significant difference between the working memory function of the experimental group with Chinese language difficulties and that of the experimental group with typical Chinese language abilities [*F*(1,89) = 16.26, *p* = 0.000>0.01], indicating that the inhibitory function of students with Chinese language difficulties was significantly lower than that of their peers before the experiment. At the mid-test, no significant difference was observed between the inhibitory function of the experimental group with language difficulties and that of the typical experimental group [*F*(1,89) = 0.02, *p* = 0.887>0.05], indicating that the 5-week exercise intervention improved the inhibitory function of students with language difficulties more effectively than that of typical students; At the post-test, no significant difference was found in the working memory function between the experimental group with language difficulties and the typical group [*F*(1,89) = 0.01, *p* = 0.931 >0.05], indicating that the 6–10 week exercise intervention improved the working memory function of elementary students with language difficulties more effectively than that of typical students (Figure 8). The results demonstrate that the 10-week exercise intervention improved the working memory function of elementary students with language difficulties more effectively than it did in typical students.

##### 3.4.3.3 Comparison of metacognitive function between experimental group students with language difficulties and typically developing elementary students at three time points

**FIGURE 9:**
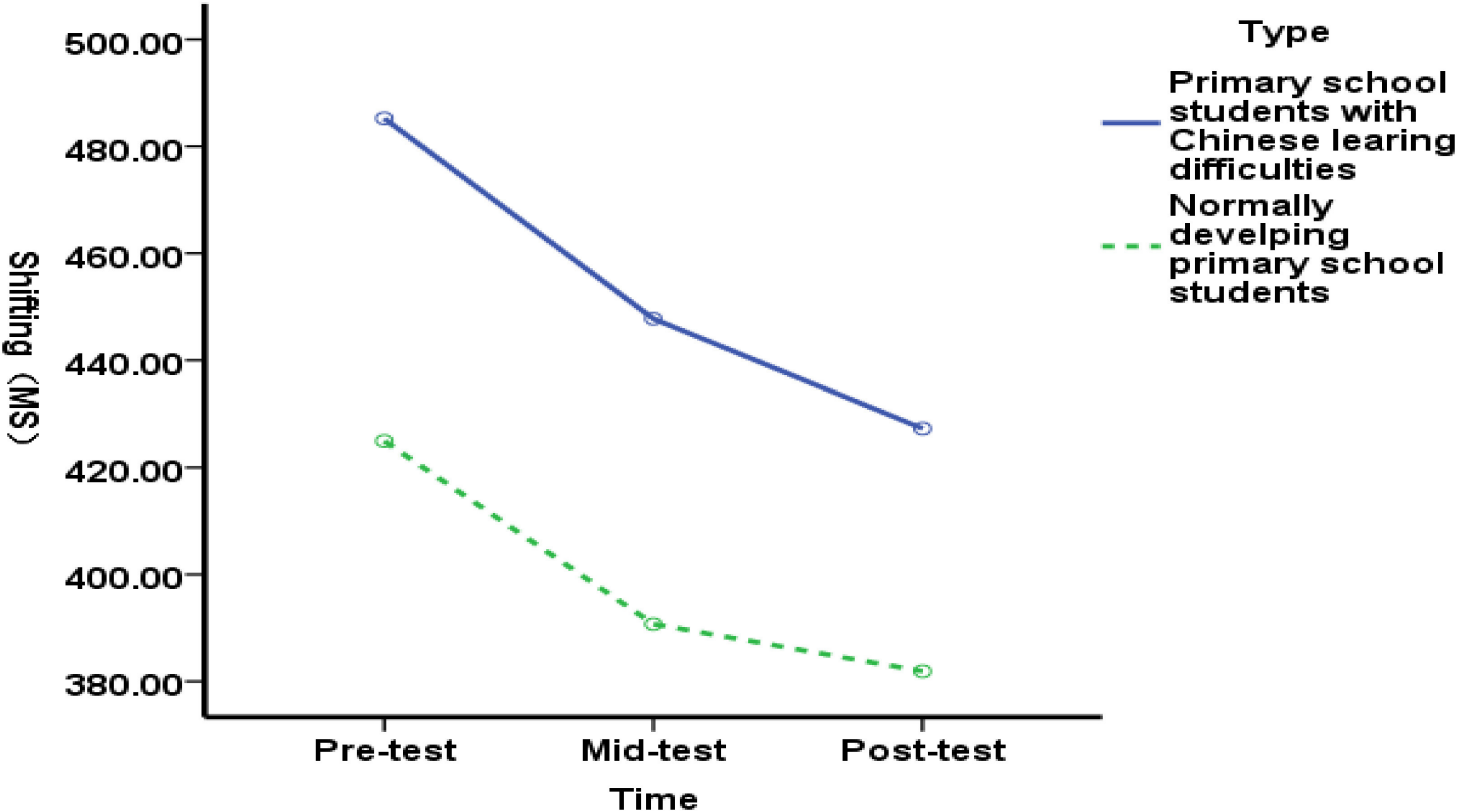
Changes in transitional functions among Chinese language-impaired and typically developing elementary students before, during, and after the exercise intervention.

Figure 9 shows that, Pre-test comparisons revealed no significant difference between the shifting functions of the experimental group’s Chinese-language-impaired elementary students and those of the experimental group’s typically developing elementary students [*F*(1, 89) = 3.07, *p* = 0.083>0.05], indicating that both groups exhibited comparable shifting function levels. At the mid-test, no significant difference was found between the shifting function of the experimental group with language difficulties and that of the experimental group without language difficulties [*F*(1, 89) = 3.82, *p* = 0.053>0.05]; At the post-test, no significant difference was found between the shifting function of the experimental group with language difficulties and that of the experimental group without language difficulties [*F*(1, 89) = 3.05, *p* = 0.084>0.05]. Results indicate that the 10-week exercise intervention did not improve shifting function in either the experimental group with language difficulties or the experimental group without language difficulties.

## 4 Discussion

### 4.1 Effects of exercise intervention on executive function in elementary students with language learning difficulties and typically developing peers

This study found that the developed “fun games + creative jump rope + creative running” exercise intervention program can enhance working memory in both Chinese language-learning-impaired and typical elementary school students. The improvement in working memory function was as follows: 10-week exercise intervention level > 5-week exercise intervention level > pre-intervention level. This suggests that physical exercise can enhance executive function in both Chinese language-learning-impaired and typical elementary school students. The “fun games + creative jump rope + creative running” exercise intervention enhances working memory in both language-impaired and typical elementary students through the following pathways.

First, the exercise program promotes improvements in elementary students’ working memory. The “fun games + creative jump rope + creative running” exercise program is specifically designed to develop working memory. The movement sequences emphasize students actively storing the actions required for the current task and then retrieving the necessary actions for new tasks based on what they have already learned. To develop elementary students’ working memory, fun games emphasize changing rules and activities, creative jump rope focuses on novel techniques and organizational forms, and creative running highlights new running styles and practice methods. Designed for elementary students’ physical and mental characteristics, these fun games, creative jump rope, and creative running combine elements of fun, competition, and variety. Not only do they individually enhance working memory, but combining all three components further develops it.

Secondly, exercise duration is a crucial factor in enhancing working memory functional development among elementary school students. This study reveals the impact of the “fun games + creative jump rope + creative running” exercise program on the functional development of students with language-learning difficulties and their peers, as well as its dynamic characteristics over time. This aligns with previous findings. Yin et al. (2014) conducted a 20-week longitudinal intervention using “patterned running” and “martial arts + jump rope + figure-eight running” with elementary students. Results showed that 20-week students’ executive function scores were higher than those of 10-week students, who in turn were higher than pre-intervention scores. The conclusion was that both intervention programs positively promoted executive function in elementary students, with effects becoming more pronounced over time (Yin et al., 2014). The psychological mechanism by which exercise intervention improves elementary students’ working memory involves 10 weeks of fun games, creative jump rope, and creative running, alleviating academic stress, altering brain activation patterns, enhancing the dynamic regulation of working memory information, and ultimately boosting working memory function.

Finally, moderate-intensity exercise promotes the development of executive functions in elementary school students. Previous studies have shown that moderate intensity yields the most significant improvements in executive functions among elementary school students (Anonymous, 2014; Ellemberg and St-Louis-Déschênes, 2010; Tomporowski et al., 2008). Therefore, the exercise program combining “fun games + creative jump rope + creative running” employs varying exercise densities, frequencies, and organizational formats to maintain moderate-intensity levels throughout the sessions.

### 4.2 Differences in the effects of exercise interventions on executive function in elementary school students with language learning difficulties and their peers

Research also found that the “fun games + creative jump rope + creative running” exercise intervention program demonstrated superior tracking effects on the working memory function of elementary students with Chinese language difficulties compared to their peers without such difficulties. The reasons for the improved working memory function among students with Chinese language difficulties are as follows. On one hand, students with language learning difficulties typically experience greater psychological pressure during regular studies. During physical education classes, teachers use fun games, creative jump-rope activities, and running exercises to alleviate this pressure. These students experience more stress than their peers, which gradually increases positive emotions, in turn promoting the development of their cognitive functions. On the other hand, the “Fun Games + Creative Jump Rope + Creative Running” program was specifically designed to address the underdeveloped working memory function in students with language learning difficulties. Its cognitive load aligns with their cognitive memory capacity (Liu, 2004; Lyu, 2013). By repeatedly applying executive functions to complete the tasks of fun games, creative jump rope, and creative running, these students enhanced their working memory function. The improvement in working memory function among typical students was smaller than that among students with Chinese learning difficulties. This is primarily because typical students already possessed relatively strong working memory function prior to the intervention. Although improvements occurred, the potential for further enhancement was limited, indicating the emergence of a typical plateau effect.

## 5 Conclusion

The “fun games + creative jump rope + creative running” exercise intervention developed in this study positively impacted the cognitive function of both Chinese language learners with learning difficulties and typically developing elementary students, with improvements becoming more pronounced as the intervention duration increased.

The cognitive function improvements observed in the experimental group of Chinese language learners with learning difficulties were greater than those seen in the experimental group of typically developing elementary students.

## Data Availability

The original contributions presented in this study are included in the article/supplementary material, further inquiries can be directed to the corresponding author.
